# Tristetraprolin limits age-related expansion of myeloid-derived suppressor cells

**DOI:** 10.3389/fimmu.2022.1002163

**Published:** 2022-10-03

**Authors:** Kyu Hwan Kwack, Lixia Zhang, Elliot D. Kramer, Ramkumar Thiyagarajan, Natalie A. Lamb, Yukitomo Arao, Jonathan E. Bard, Kenneth L. Seldeen, Bruce R. Troen, Perry J. Blackshear, Scott I. Abrams, Keith L. Kirkwood

**Affiliations:** ^1^ Department of Oral Biology, University at Buffalo, Buffalo, NY, United States; ^2^ Department of Oral Microbiology, College of Dentistry, Kyung Hee University, Seoul, South Korea; ^3^ Department of Medicine, University at Buffalo, Buffalo, NY, United States; ^4^ Departments of Immunology, Roswell Park Comprehensive Cancer Center, Buffalo, NY, United States; ^5^ Division of Geriatrics and Palliative Medicine, University at Buffalo, Buffalo, NY, United States; ^6^ Research Service, Veterans Affairs Western New York Healthcare Service, Buffalo, NY, United States; ^7^ Department of Biochemistry, University at Buffalo, Buffalo, NY, United States; ^8^ Genomics and Bioinformatics Core, New York State Center of Excellence for Bioinformatics and Life Sciences, University at Buffalo, The State University of New York, Buffalo, NY, United States; ^9^ Signal Transduction Laboratory, National Institute of Environmental Health Sciences, Research Triangle Park, NC, United States; ^10^ Departments of Biochemistry & Medicine, Duke University Medical Center, Durham, NC, United States; ^11^ Head & Neck/Plastic & Reconstructive Surgery, Roswell Park Comprehensive Cancer Center, Buffalo, NY, United States

**Keywords:** aging, tristetraprolin, myeloid-derived suppressor cells, hematopoietic progenitor cell, myeloid cell, transcriptome, bone marrow

## Abstract

Aging results in enhanced myelopoiesis, which is associated with an increased prevalence of myeloid leukemias and the production of myeloid-derived suppressor cells (MDSCs). Tristetraprolin (TTP) is an RNA binding protein that regulates immune-related cytokines and chemokines by destabilizing target mRNAs. As TTP expression is known to decrease with age in myeloid cells, we used TTP-deficient (TTPKO) mice to model aged mice to study TTP regulation in age-related myelopoiesis. Both TTPKO and myeloid-specific TTPKO (cTTPKO) mice had significant increases in both MDSC subpopulations M-MDSCs (CD11b^+^Ly6C^hi^Ly6G^-^) and PMN-MDSCs (CD11b^+^Ly6C^lo^Ly6G^+^), as well as macrophages (CD11b^+^F4/80^+^) in the spleen and mesenteric lymph nodes; however, no quantitative changes in MDSCs were observed in the bone marrow. In contrast, gain-of-function TTP knock-in (TTPKI) mice had no change in MDSCs compared with control mice. Within the bone marrow, total granulocyte-monocyte progenitors (GMPs) and monocyte progenitors (MPs), direct antecedents of M-MDSCs, were significantly increased in both cTTPKO and TTPKO mice, but granulocyte progenitors (GPs) were significantly increased only in TTPKO mice. Transcriptomic analysis of the bone marrow myeloid cell populations revealed that the expression of CC chemokine receptor 2 (CCR2), which plays a key role in monocyte mobilization to inflammatory sites, was dramatically increased in both cTTPKO and TTPKO mice. Concurrently, the concentration of CC chemokine ligand 2 (CCL2), a major ligand of CCR2, was high in the serum of cTTPKO and TTPKO mice, suggesting that TTP impacts the mobilization of M-MDSCs from the bone marrow to inflammatory sites during aging *via* regulation of the CCR2-CCL2 axis. Collectively, these studies demonstrate a previously unrecognized role for TTP in regulating age-associated myelopoiesis through the expansion of specific myeloid progenitors and M-MDSCs and their recruitment to sites of injury, inflammation, or other pathologic perturbations.

## Introduction

In both mice and humans, the aging process is accompanied by increased myelopoiesis, resulting in enhanced frequencies and numbers of hematopoietic stem and progenitor cells (HSPCs), common myeloid progenitors (CMPs), granulocyte-monocyte progenitors (GMPs) and mature myeloid cells (1–5). Enhanced myelopoiesis correlates with an increased frequency of myeloid leukemias and the production of myeloid-derived suppressor cells (MDSCs) (6, 7). Hence, enhancing an understanding of the molecular mechanisms of an age-associated myeloid-biased expansion has potential clinical significance in myeloid-driven inflammation, disorders, or pathologies.

With aging, there is an increased production of inflammatory cytokines and chemokines systemically referred to as inflammaging (8). Key inflammatory cytokines, including interleukin (IL)-1, IL-6 and tumor necrosis factor-alpha (TNF-α), are expressed by HSPCs, and engagement of these inflammatory mediators can stimulate myelopoiesis and inhibit lymphopoiesis (9). Aged HSPCs express a proinflammatory gene signature consistent with the concept that the inflammatory microenvironment contributes to the age-related changes in hematopoiesis (10). These cell-extrinsic events may initiate HSPC population shifts within the bone marrow to elicit expansion in downstream myeloid progenitors. Thus, a better understanding of the cell-intrinsic systems that are engaged may provide new insights into the mechanisms of age-related increases in myelopoiesis.

Tristetraprolin (TTP), encoded by zinc finger protein 36 (*Zfp36*), is an RNA binding protein essential for enhancing the degradation of target mRNAs, including the key inflammatory cytokines TNF-α, IL-2, and IL-6 (11–13). TTP-deficient (TTPKO) mice exhibit severe inflammatory phenotypes such as cachexia, erosive arthritis, conjunctivitis, dermatitis, periodontitis and myeloid hyperplasia, although they appear to be normal at birth (14, 15). Unlike some other key RNA binding proteins, TTP expression declines with advancing age in humans, particularly in cells within the immune system (16). Myeloid-specific TTP-deficient (cTTPKO) mice, which ablates TTP from myeloid cells, did not show severe inflammation, despite being highly vulnerable to low-dose LPS (17). In contrast, gain-of-function TTP knock-in (TTPKI) mice, which exhibit modest TTP overexpression from its endogenous locus, exhibited protection against several inflammatory diseases (18). Thus, TTP plays a significant role in the control of mRNAs that regulate myeloid cell inflammatory signatures which may have profound systemic ramifications.

MDSCs contribute to acute and chronic inflammatory processes associated with aging (19–21), and have been recurrently detected in different inflammatory-based pathologic disorders. The current predominant view is that MDSCs, comprised of two of the major subsets, M-MDSCs (CD11b^+^Ly6C^lo^Ly6G^+^) and PMN-MDSCs (CD11b^+^Ly6C^hi^Ly6G^−^), differentiate along the same hematopoietic pathways as monocytes and neutrophils, and their expansion is controlled by increased production of GM-CSF, CSF-1, G-CSF, and other growth factors generated during inflammatory processes (22). These pathways involve pluripotent HSPCs, multipotent CMPs, and oligo- or bipotential GMPs. HSPCs give rise to progeny that progressively lose self-renewal capacity and become restricted to one lineage. The points at which HSPCs commit to each of the various lineages remain mostly unknown. In this study, we investigated the impact of age-related decline of TTP expression on myelopoiesis, and the generation and expansion of the resultant MDSCs.

Although early studies with global loss of TTP showed increased levels of myelopoiesis (14), an in-depth examination of the HSPCs and a detailed analysis of the myeloid precursor population shifts within the bone marrow have not been performed. Here, we examined the loss of TTP that is associated with aging to understand how age-related TTP declines may play a role in myelopoiesis and MDSC expansion and the associated inflammaging phenotype. Through integration of single-cell transcriptomics from whole bone marrow, coupled with comprehensive flow cytometric analyses of myeloid progenitors, we sought to understand TTP-directed transcriptomic changes in myeloid cell subsets that result in age-related MDSC expansion.

## Materials and methods

### Mice

All mice on a C57BL/6 background were maintained and housed under specific pathogen free (SPF) conditions. Mice were housed in positive ventilated cages, fed autoclaved standard chow diet, and provided bedding and enrichments such as nestlets and enviro-dry. All mice were kept in a controlled temperature and environment under a 12 h light/12 h dark cycle. The young (6-month-old) and old (24-month-old) male mice were acquired from National Institute on Aging (NIA) in Charles River Laboratories (Wilmington, MA). Wild type (WT) and *Zfp36^-/-^* (TTPKO) mice were generated by breeding heterozygous dams (14). Newborn mice were tail snipped to determine the genotype using WT- and TTP KO-specific primers: Fwd-5’-GGCCGAAGCTGTGCTGGGT-3’ and Rev-5’CTGGCCAGGGAGAGCTAGGTC-3’ (Eurofins MWG Operon). TTPKI mice were established as knock-in mice, in which TTP mRNA is stabilized under physiological conditions to modestly increase TTP mRNA and protein levels (18). The generated mice were genotyped using the following primers: 5′-CGTCTCCCCATCTTCAATCGT-3′ and 5′-CAACCCCCCCCAAAAAATAGA-3′. Myeloid-specific TTP-deficient (cTTP KO) mice were generated by crossing *loxP*-flanked *Zfp36* mice (*Zfp36* ^flox/flox^) with LysM-cre mice (17). Mice were genotyped using primers: 5’-CCCAGAAATGCCAGATTACG-3’, 5’-CTTGGGCTGCCAGAATTTCTC-3’ and 5’-TTACAGTCGGCCAGGCTGAC-3’. All experiments with mice were approved by the Institutional Animal Care and Use Committee at the University at Buffalo and performed according to the National Institutes of Health Guide for Care and Use of Laboratory Animals (ORB15087Y; expiration November 30, 2023).

### Isolation of tissue cells

Bone marrow cells were obtained by flushing femurs from mice with RPMI 1640 (Corning Inc., Corning, New York, NY, USA) medium. Mesenteric lymph nodes (mLNs) and spleen were dissected and minced in RPMI 1640 medium. Single cell suspensions were obtained by passing cell suspensions through a 70μm filter. Bone marrow cells and splenocytes were treated with ACK lysis buffer (Gibco, Invitrogen, USA).

### Antibodies and flow cytometry

For flow cytometry, Fc receptors were blocked by treating with Fc block (BD) before staining with other surface markers. To characterize the phenotype of bone marrow cells, we stained for various anti-mouse antibodies (Abs) (**Table S1**). For surface staining, the cells were incubated with Abs for 30 min. After staining, cells were washed, and fixed in 4% formaldehyde (Sigma Aldrich).

For the bone marrow progenitor analysis, one million cells were suspended in staining buffer (dPBS + 0.5% BSA + 2mM EDTA) and directly conjugated Abs were used for staining (**Table S1**). Stained samples were treated with DAPI (ThermoFisher Scientific) to exclude dead cells. Samples were read on the LSR II flow cytometer (BD Biosciences) *via* FACSDiva version 6.1.3 software. Data analysis was performed using FCS Express 7.0 according to the bone marrow progenitor markers previously described (23). To represent the progenitor population as TriMap, DownSample (v.3.3.1), TriMap (v.0.2) FlowJo plugins were used (FlowJo LLC). Both male and female mice were used in these experiments.

### T-lymphocyte proliferation assay

MDSCs were isolated from the bone marrow by immuno-magnetic bead selection methods using the Myeloid-Derived Suppressor Cell Isolation Kit (Miltenyi Biotec). CD3^+^ T cells were isolated using CD3 MicroBead kit (Miltenyi Biotec) from the spleens of wild-type C57BL/6 mice and stained with CellTrace Violet (CTV) Cell Proliferation Kit (Invitrogen). CTV-labeled CD3^+^ T cells were plated and activated by anti-CD3 Ab (3 µg/ml) and an anti-CD28 Ab (5µg/ml, Thermo Fisher Scientific). The isolated MDSCs were co-cultured with activated T cells for 3 days. The cells were washed, collected, and stained with CD4-PE-cy7 (e-Bioscience) and CD8-APC-cy7 (Miltenyi Biotec). All samples were acquired on MACSQuant System (Miltenyi Biotec) and analyzed with FlowJo v10.8 software.

### Bulk RNA sequencing

Total mRNA was obtained from CD11b^+^Ly6C^hi^Ly6G^-^ M-MDSCs isolated from young and old mice using RNeasy Mini Kits (Qiagen, USA) according to the manufacturer’s instruction. A NovaSeq 6000 System (Illumina) was used to perform whole-genome sequencing. Per-cycle basecall (BCL) files generated by the NovaSeq 6000 were converted to per-read FASTQ files using bcl2fastq version 2.20.0.422, using default parameters. The quality of the sequencing was reviewed using FastQC version 0.11.9. Detection of potential contamination was done using FastQ Screen version 0.14.1. FastQC and FastQ Screen quality reports were summarized using MultiQC version 1.9.0.

Genomic alignments were performed using HISAT2 version 2.2.1 using default parameters. UCSC reference mm10 was use for the reference genome and gene annotation set. Sequence alignments were compressed and sorted into binary alignment map (BAM) files using samtools version 1.15.1. Counting of mapped reads for genomic features was performed using Subread featureCounts version 2.0.0 using the parameters -s 2 –g gene_name –t exon –Q 60 -B -C, and the annotation file specified with –a was the UCSC mm10 reference provided by Illumina’s iGenomes. Alignment statistics and feature assignment statistics were again summarized using MultiQC.

Differentially expressed genes were detected using the Bioconductor package DESeq2 version 1.32.0. Alpha was set to 0.05. DESeq2 tests for differential expression used negative binomial generalized linear models, dispersion estimates, and logarithmic fold changes.

### Preparation of single-cell expression reference dataset

To compare TTP expression in young and aged mouse models, reference scRNA-seq data was processed from the Tabula Muris Senis (TMS) dataset (24, 25). Droplet scRNA-seq data objects were downloaded and loaded into R studio V 4. The bone marrow droplet data was subset based on cell populations that make up the myeloid compartment, including, basophils, granulocytes, granulocytopoietic cells, macrophages, monocytes, and promonocytes. The resulting cell population was analyzed using the default Seurat workflow. *Zfp36* expression as a function of age was evaluated using the Seurat function violin plot.

### Single-cell RNA sequencing

Soft tissue was dissected from femurs of three-month-old TTP myeloid-specific or global KO mice along with controls on a C57BL/6 background. Long-bone BM cells were flushed from the femurs with RPMI 1640 (Corning Inc., Corning, New York, NY, USA) medium. Femurs were then placed in 1.5ml microfuge tubes supported by 0.5ml microfuge and centrifuge at 8000 rpm for 10 minutes. The bone marrow pellet was resuspended 5ml of RPMI 1640 culture medium. A single cell suspension was obtained by passing 18-, 21- and 25-gauge needles in sequence.

Cell suspensions isolated from femurs were captured on the 10X Genomics Chromium Controller instrument (10X Genomics). The libraries were prepared according to the manufacturer’s protocol (10X 3’ Expression Kit, version 3; 10X Genomics), and loaded onto the illumina NovaSeq using the S1 Flowcell in high-output mode with a typical target of 30,000 reads per cell. Post-sequencing, data were demultiplexed and provided as input to the 10X Genomics Cell Ranger pipeline (version 5), which quantified the transcriptomic profile of each cell through alignment to the mouse reference genome (GRCm38/mm10). The Cell Ranger matrix files were then used as input to the R Seurat package version 4 (26).

Mapping rates to the reference genome were greater than 95% for all samples. Approximately 16,140 cells from the femurs of WT [previously sequenced in (25)], 15,996 cells from the femurs of cTTPKO, and 15,730 cells from the femurs of TTPKO mice were sequenced. Cells with abnormal gene detection rates (< 200 and > 6000) and high mitochondrial transcriptional load (> 15%), an indicator of cellular stress, were reported as outliers and filtered out of the analysis. Data were subjected to Seurat normalization followed by principal component analysis (PCA) and Uniform Manifold Approximation and Projection (UMAP) dimensionality reduction, and Shared Nearest Neighbor (SNN) graph to cluster cells with similar transcriptomic profiles.

Clusters were annotated *via* scCATCH, an automated platform for identifying cell types (27). After scCATCH provided a basis for identifying cell types, we manually reviewed the marker genes in individual clusters and performed further sub-clustering for cell type labeling. To investigate M-MDSCs, we subset and re-clustered the monocyte population. M-MDSCs were defined through a genetic panel (*Cxcr2, S100a9, S100a8, Ifitm1, Lrg1, Stfa2l1, Retnlg, Il1b, BC100530, Gm5483*), previously determined thorough scRNA-seq (28). The M-MDSC population isolated to one distinct cluster and was defined as the cluster with the highest expression of the M-MDSC gene panel.

Cells were subset that give rise to MDSCs to further investigate the effect of TTP knockout on progenitor populations. The resulting cell population was independently processed using the primary Seurat workflow in order to detect nuances within the myeloid compartment. This allowed for more specific labeling of progenitor cell types. CMP, GMP, and MEP populations clustered in the center and informed labeling of cells upstream in development, including cMoP/MPs and GPs.

### Serum enzyme-linked immunosorbent assay

For serum collection, blood samples were collected from young and old mice by cardiac puncture, incubated at room temperature for 30 minutes, and centrifuged at 1,000 x g for 10 minutes. Serum levels of CCL2, CXCL2, IL-6, and TNF-α were quantified using an automatic ELISA (ELLA, Protein Simple) according to the manufacturer’s instructions.

### Statistical analysis

Statistical analysis was done with GraphPad Prism 8.4 (GraphPad Software Inc., La Jolly, CA) with unpaired Student t tests, or one and two-way analysis of variance (ANOVA) followed by Tukey’s multiple comparisons test. For scRNA-seq, per-cell gene expression was log-normalized using the Seurat function NormalizeData with default parameters, followed by data scaling using ScaleData. Differential expression testing was performed using the Wilcoxon Rank Sum test, using Seurat’s FindMarkers and FindAllMarkers functions with default parameter sets. All reported data of P-values less than 0.05 were considered statistically significant.

## Results

### Myeloid cell populations expand as Zfp36 expression declines with aging

**Figure 1:**
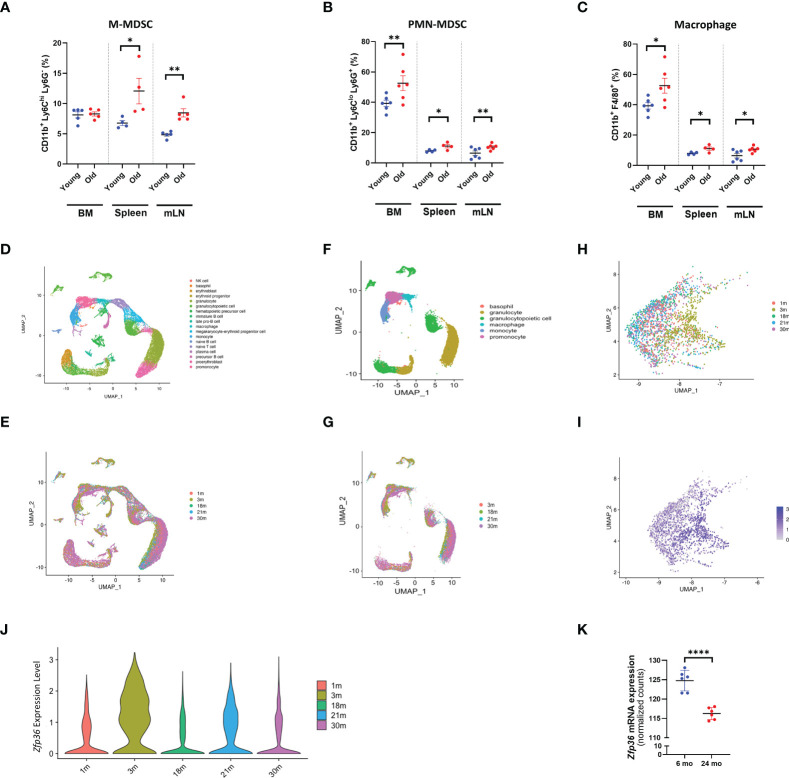
With aging, the myeloid population expands and *Zfp36* expression is downregulated. **(A)** Flow cytometry analysis of M-MDSC populations in bone marrow, spleen, and mesenteric lymph nodes from young and old mice. **(A)** The UMAP plot of cells isolated from bone marrow. **(B)** Flow cytometry analysis of PMN-MDSC populations in bone marrow, spleen, and mesenteric lymph nodes from young and old mice. **(C)** Flow cytometry analysis of macrophage populations in bone marrow, spleen, and mesenteric lymph nodes from young and old mice. **(D)** The UMAP plot of cells isolated from bone marrow from 3-month-old mice. **(E)** The UMAP plot of 1, 3, 18, 21, and 30-month datasets shows the distribution of each age group. **(F)** The UMAP plot of myeloid cells isolated from bone marrow. **(G)** The UMAP plot of 1, 3, 18, 21, and 30-month datasets shows the distribution of each age group. **(H, I)** The UMAP plot of 1, 3, 18, 21, and 30-month datasets shows the distribution of *Zfp36* expression in myeloid cells of each age group. **(J)** Violin plots showing the expression of *Zfp36* in myeloid cells of each age group. **(K)** Quantification of *Zfp36* mRNA expression in M-MDSC populations of young and old mice based on bulk RNA sequencing. Comparisons were analyzed using unpaired t tests; data are presented as mean ± SEM, *P < 0.05, **P < 0.01, ****P < 0.0001.

To explore quantitative changes in the myeloid compartment in relation to aging, we evaluated both primary and secondary lymphoid tissues, namely the bone marrow, spleen, and mesenteric lymph nodes (mLNs) of young (6-month-old) and old (24-month-old) mice. In aged bone marrow, no changes in M-MDSCs were observed, whereas PMN-MDSCs and macrophages were significantly expanded relative to bone marrow of young mice. All myeloid cell populations analyzed were expanded in peripheral (secondary) lymphoid tissues of the spleen and mLNs of aged mice (**Figure 1A-C**).

To determine whether the expression of TTP decreases with aging in mice as in humans, we explored Tabula Muris Senis, a comprehensive single-cell transcriptomic data atlas from *Mus musculus* comprising more than 500,000 cells from 18 organs and tissues across the mouse lifespan (from 1 month to 30 months of age) (24). Transcriptomic signatures of total bone marrow from 3-month-old mice were obtained from reference data, and 18 distinct cell subpopulations were identified (**Figure 1D**). As seen in the UMAP, there was a marked difference in the transcriptomic signature of total bone marrow according to age (**Figure 1E**). To focus on the myeloid compartment, only the corresponding portion in UMAP was selected (**Figure 1F**), and the distribution of the myeloid compartment clearly exhibited a shift with age (**Figure 1G**). Myeloid compartments were selected and re-clustered to represent UMAP by age, and it was confirmed that they were shifted according to age (**Figure 1H**). The expression level of *Zfp36* was confirmed in UMAP, and it was also confirmed that the expression of *Zfp36* decreased with age except at one-month of age (**Figure 1I**), likely due to the immature immune system. Violin plots confirmed that the expression of *Zfp36* was highest at 3 months of age and decreased with aging (**Figure 1J**). Since to our knowledge there are no reports of *Zfp36* expression in MDSC populations, we compared *Zfp36* expression in M-MDSCs, and, like other myeloid cells, *Zfp36* expression in M-MDSCs decreased with age (**Figure 1K**). However, *Zfp36* expression in PMN-MDSCs was not assessed. These results indicate that *Zfp36* expression is reduced in the myeloid population of aged mice, suggesting the possibility that mice deficient in TTP encoded by *Zfp36* can be used as a model system for aged mice.

### TTP deficiency increases myeloid populations except for M-MDSCs within the bone marrow

**Figure 2:**
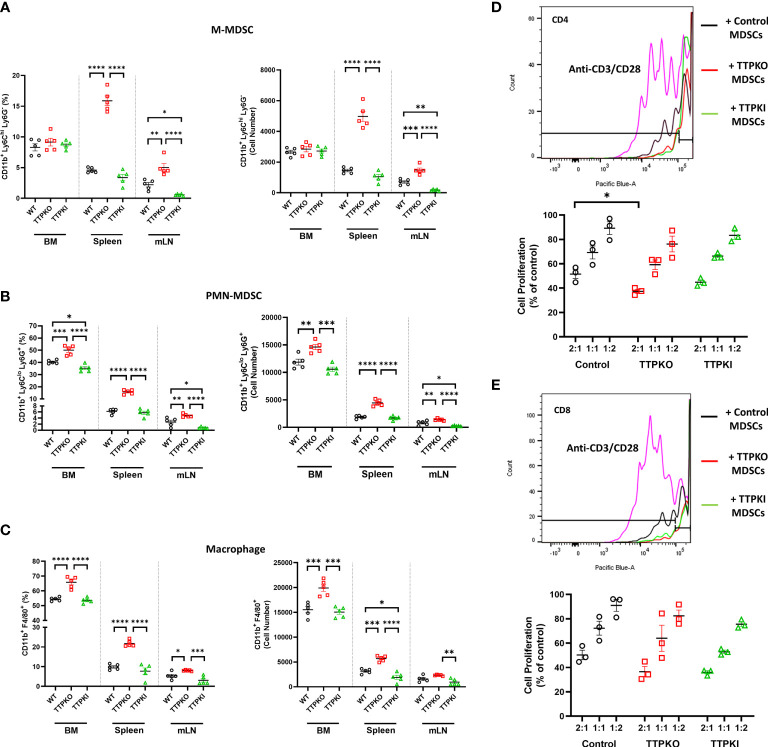
M-MDSCs are expanded except in the bone marrow of TTPKO mice. **(A)** Flow cytometry analysis of M-MDSC populations in bone marrow, spleen, and mesenteric lymph nodes from WT control, TTPKO, and TTPKI mice. **(B)** Flow cytometry analysis of PMN-MDSCs in bone marrow, spleen, and mesenteric lymph nodes from WT control, TTPKO, and TTPKI mice. **(C)** Flow cytometry analysis of macrophage populations in bone marrow, spleen, and mesenteric lymph nodes from WT control, TTPKO, and TTPKI mice. **(D)** Proliferated CD4^+^ T cell with anti-CD3/CD28 are shown in pink. CD4^+^ T cell proliferation assay of MDSCs isolated from WT control (black), TTPKO (red), and TTPKI (green) mice. **(E)** Proliferated CD8^+^ T cell with anti-CD3/CD28 are shown in pink. CD8^+^ T cell proliferation assay of MDSCs isolated from WT control (black), TTPKO (red), and TTPKI (green) mice. Statistical comparisons were performed with one-way analysis of variance with Tukey’s multiple comparisons test; data are presented as mean ± SEM, *P < 0.05, **P < 0.01, ***P < 0.001, ****P < 0.0001.

To determine if the lack of TTP expression alters the myeloid compartment, as in aged mice, we evaluated the bone marrow, spleen and mLNs using both loss-of-function global TTPKO and gain-of-function TTPKI models, compared to the control WT mice (**Figure 2**). The frequency of M-MDSCs was significantly higher compared to the WT controls in both the spleen and mLNs, but not in the bone marrow of TTPKO mice. In TTPKI mice, M-MDSC frequency was significantly lower compared to WT controls in the mLNs, but not in the bone marrow and spleen (**Figure 2A**). Instead, in TTPKO mice, PMN-MDSCs and macrophages were significantly increased in both the spleen, mLNs and bone marrow, whereas in TTPKI mice, only PMN-MDSCs were significantly reduced in the bone marrow and mLNs (**Figures 2B-C**). Similar alterations of these myeloid populations were observed in female mice indicating that genotype and not sex-related differences, is the likely primary influence on the observed changes in the myeloid populations. (**Figure S2**).

Detailed flow cytometric analyses were performed in an effort to help distinguish M-MDSCs from other myeloid cells, including inflammatory monocytes. There was no significant change in inflammatory monocytes, whereas M-MDSCs significantly expanded in the spleen in TTP-deficient hosts (**Figure S3**). To show that M-MDSCs had bona fide immunosuppressive activity, we performed functional tests in T lymphocyte proliferation assays. MDSCs isolated from WT, TTPKO, and TTPKI mice significantly inhibited CD4^+^ or CD8^+^ T lymphocyte proliferation, confirming that they were indeed MDSCs (**Figure 2D-E**). M-MDSCs from global TTP KO exhibited increased immunosuppressive activity with CD4^+^ T-cells at 2:1 (M-MDSCs: CD4^+^) ratio compared to control. These data suggest that the global loss of TTP results in the expansion of myeloid populations, although M-MDSCs are not expanded in the bone marrow, similar to what is observed in bone marrow of aged mice.

### Myeloid-specific TTP deficiency increases myeloid populations in the spleen and mLN, but not in the bone marrow

**Figure 3:**
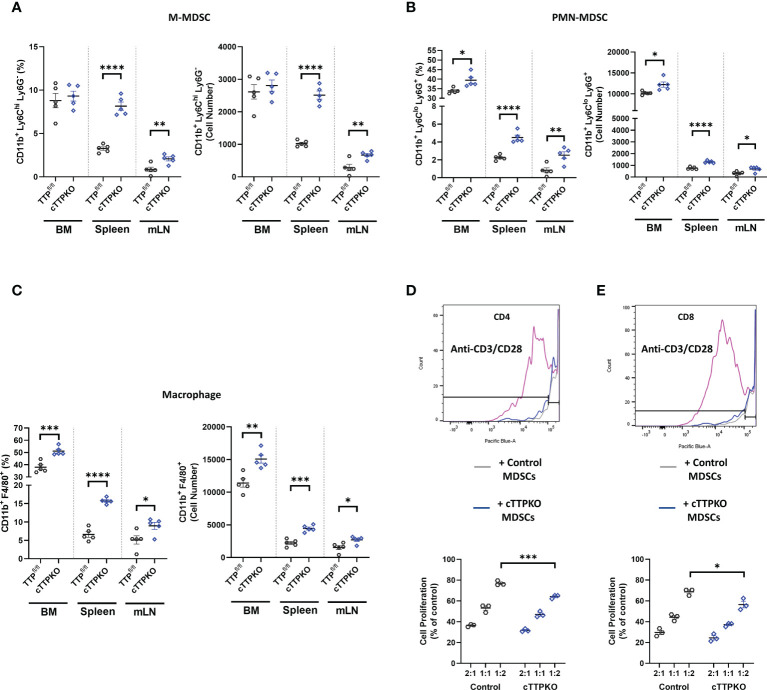
M-MDSCs are expanded except in the bone marrow of cTTPKO mice. **(A)** Flow cytometry analysis of M-MDSC populations in bone marrow, spleen, and mesenteric lymph nodes from control and cTTPKO mice. **(B)** Flow cytometry analysis of PMN-MDSCs in bone marrow, spleen, and mesenteric lymph nodes from control and cTTPKO mice. **(C)** Flow cytometry analysis of macrophage populations in bone marrow, spleen, and mesenteric lymph nodes from control and cTTPKO mice. **(D)** Proliferated CD4^+^ T cell with anti-CD3/CD28 are shown in pink. CD4^+^ T cell proliferation assay using MDSCs isolated from control (grey) and cTTPKO (blue) mice. **(E)** Proliferated CD8^+^ T cell with anti-CD3/CD28 are shown in pink. CD8^+^ T cell proliferation using MDSCs isolated from control (grey) and cTTPKO (blue) mice. One-way analysis of variance with Tukey’s multiple comparisons test and unpaired *t* tests were used; data are presented as mean ± SEM, *P < 0.05, **P < 0.01, ***P < 0.001, ****P < 0.0001.

To examine cell-intrinsic effects of TTP in myelopoiesis, we made use of myeloid-deficient TTP mice (LysMcre/*Zfp36* ^flox/flox^ or M-TTP KO) compared to the *Zfp36* floxed control mice (*Zfp36* ^flox/flox^), focusing on the bone marrow, spleen, and mLNs from 3-month-old male mice (**Figure 3**). Similar to TTPKO, M-MDSCs percentages were not significantly different in the bone marrow, but were significantly increased in both the spleen and mLNs (**Figure 3A**). However, PMN-MDSCs (**Figure 3B**) and macrophages (**Figure 3C**) were significantly increased in the bone marrow, as well as in the spleen and mLNs (**Figures 3B-C**). We also confirmed MDSC phenotype using T proliferation assays. MDSCs isolated from TTP^fl/fl^ and cTTPKO mice inhibited CD4^+^ (**Figure 3D**) or CD8^+^ (**Figure 3E**) T lymphocyte proliferation. M-MDSCs from global cTTP KO exhibited increased immunosuppressive activity with CD4^+^ and CD8^+^ T-cells at 1:2 (M-MDSCs: CD4^+^ orCD8^+^) ratio compared to control.

### TTP deficiency causes an expansion of M-MDSC progenitors in the bone marrow

**Figure 4:**
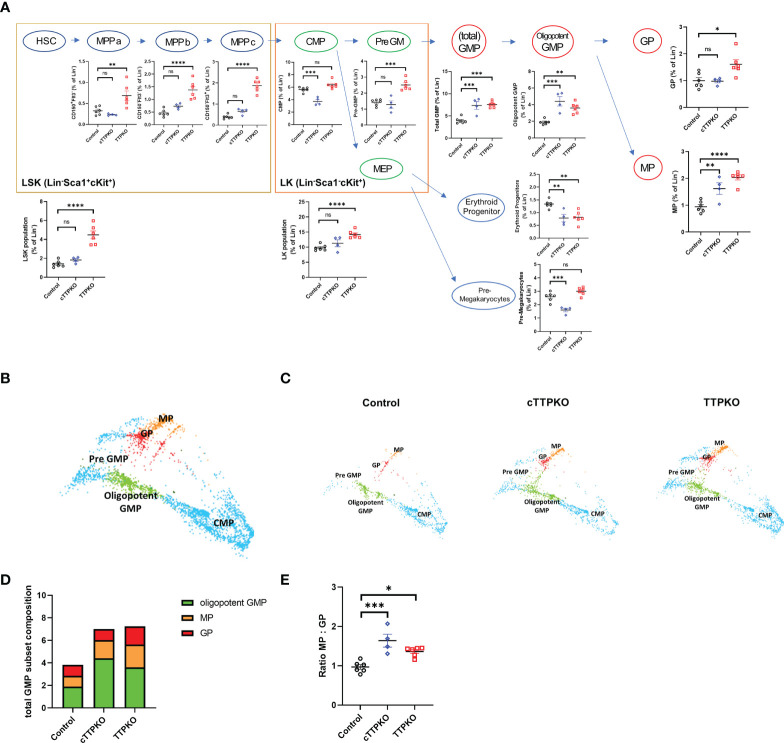
GMP and MP myeloid progenitor cells of TTPKO and cTTPKO mice are expanded. **(A)** Schematic diagram of the mouse HSPC hierarchy and flow cytometry results for the corresponding populations were combined. **(B)** Dimensional reduction utilizing TriMap was used to visualize progenitor populations. **(C)** Progenitor cells from control, cTTPKO, and TTPKO mice were individually displayed with TriMap. **(D)** Total GMP frequencies are shown as the breakdown of the different myeloid progenitor subsets. **(E)** MP : GP ratio were analyzed. One-way analysis of variance with Tukey’s multiple comparisons test and unpaired *t* tests were used; data are presented as mean ± SEM, *P < 0.05, **P < 0.01, ***P < 0.001, ****P < 0.0001.

We hypothesized that the increases in MDSCs observed in the periphery of TTPKO and cTTPKO mice depend on an enhanced level of myelopoiesis of their progenitor cells in the bone marrow. Therefore, we performed detailed flow cytometric analyses using marker panels specific for several major myeloid progenitors. The percentages for the Lin^-^ population of the LSK population and the LK population were significantly higher in TTPKO mice compared to the WT controls, whereas in cTTPKO mice, no significant differences were observed (**Figure 4A**). However, the percentages for the Lin^-^ population of total GMPs [which comprises both early- and later-stage myeloid progenitors, namely, oligopotent GMPs, granulocyte progenitors (GPs), and monocyte progenitors (MPs)] and MPs separately, considered as the antecedents of M-MDSCs, were significantly elevated in both the cTTPKO and TTPKO mice (**Figure 4A**). These results demonstrate that while M-MDSCs were not increased in the bone marrow, an increase in the progenitors of M-MDSCs was observed in cTTPKO and TTPKO mice, suggesting that TTP limits the expansion of these progenitors. We performed dimensional reduction *via* TriMap, focusing on such MPs (**Figure 4B**). As seen in TriMap projections, there were marked differences in the MPs of control, cTTPKO, and TTPKO bone marrow (**Figure 4C**). The contribution of oligopotent GMP to total GMP population expansion in cTTPKO/TTPKO mice was greatest in both cTTPKO and TTPKO genotypes (**Figure 4D**). However, the MP to GP ratio was increased in both cTTPKO and TTPKO mice, indicating that the expansion of the total GMP was likely due to the MP component (**Figure 4E**).

### Differences in single-cell atlas of bone marrow and comparative analysis of heterogeneity of M-MDSCs

**Figure 5:**
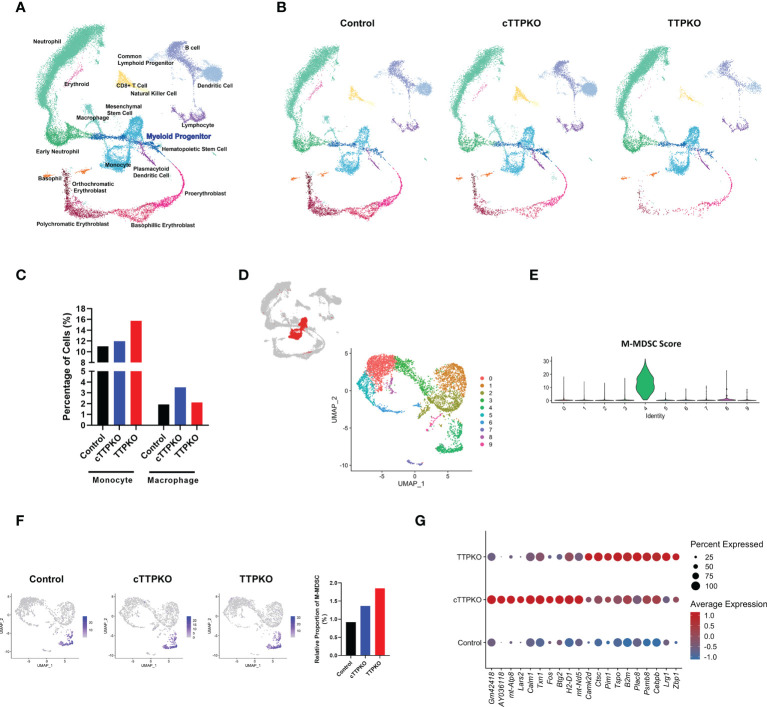
Transcriptional profiling of bone marrow-derived cells reveals distinct differentiation landscapes in bone marrow of TTPKO/cTTPKO mice as well as in M-MDSCs. **(A)** Integrated Seurat analysis of samples from total mouse bone marrow displayed in UMAP. **(B)** UMAP of control, cTTPKO, and TTPKO mice bone marrow samples displayed individually. **(C)** The percentage of neutrophils, monocytes, and macrophages from control, cTTPKO, and TTPKO mice, plotted as a percentage of total cells sequenced in each sample. **(D)** Subpopulations of monocyte clusters are shown in red, and 10 distinct sub-clusters were reconstructed and displayed as UMAP. **(E)** Expression of M-MDSC specific panel (score) is shown in violin plot. **(F)** M-MDSC populations of control, cTTPK, and TTPKO mice are individually displayed in UMAP and compared graphically. **(G)** Dot plot showing top genes in which M-MDSC populations are differentially expressed in control, cTTPKO, and TTPKO mice.

To appreciate the transcriptomic changes within bone marrow myeloid progenitor populations of global and myeloid-specific TTP-deficient mice, we performed scRNA-seq to obtain transcriptomic signatures in total bone marrow and identified 20 distinct cell subpopulations (**Figure 5A**). To investigate heterogeneity at the level of individual cells without bias, we analyzed and compared both cell population and transcriptomic differences between the samples. As seen in the UMAP, there were marked differences in the distribution of the bone marrow populations of control, cTTPKO, and TTPKO mice (**Figure 5B**). Similar to flow cytometry results, the transcriptional profiles of monocytes and macrophages were increased in cTTPKO and TTPKO mice compare to the controls (**Figure 5C**).

For detailed identification with a focus on M-MDSCs, we first classified monocyte clusters from UMAP, as previously described (25). The monocyte population was then further divided into 10 distinct sub-clusters, and the distributions of the monocyte sub-clusters also differed from one another (**Figure 5D**). Based on the expression of the M-MDSC-specific gene panel, sub-cluster 4 was identified as the M-MDSC population (**Figure 5E**). Although flow cytometry analysis did not show a significant change in the percentage of M-MDSCs, the transcriptomic profiles showed a slight increase in cTTPKO mice and a greater increase in TTPKO mice compared to the controls (**Figure 5F**). Transcriptional profiles of the M-MDSC populations were notably different from cTTPKO or TTPKO mice and controls, including *B2m*, known to be associated with osteoclast formation (29) (**Figure 5G**).

### Single-cell RNA sequence analysis revealed high expression of mobilization-related factors in M-MDSC progenitors

**Figure 6:**
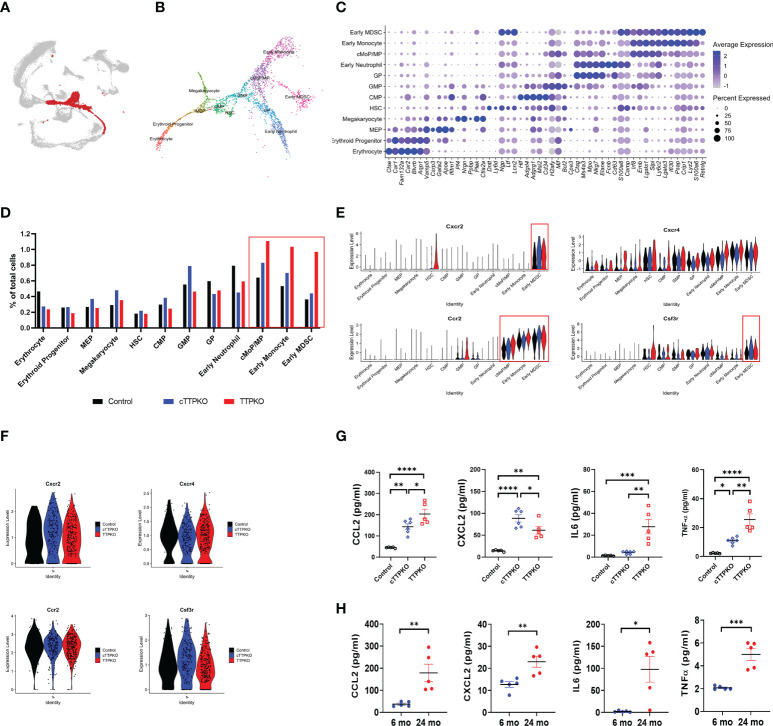
Mobilization factor *Ccr2* expression is high in M-MDSC progenitors. **(A)** Subpopulations of myeloid progenitors in bone marrow-derived cells were selected and marked in red. **(B)** Myeloid progenitors were divided into 12 distinct clusters, each labeled according to the panel of genes expressed **(C)** The panel of genes to distinguish the 12 distinct clusters and the expression level in each cluster are shown as feature plots. **(D)** scRNA-seq of relative proportions of myeloid progenitors from control, cTTPKO, and TTPKO mice. **(E)** Violin plot showing the expression of factors related to mobilization in each progenitor cluster. **(F)** Violin plot showing the expression of factors related to mobilization in M-MDSC cluster. **(G)** Quantification of chemokines in serum from control, cTTPKO, and TTPKO mice based on automatic ELISA. **(H)** Quantification of chemokines in serum from young and old mice based on automatic ELISA. One-way analysis of variance with Tukey’s multiple comparisons test and unpaired *t* test were used; data are presented as mean ± SEM, *P < 0.05, **P < 0.01, ***P < 0.001, ****P < 0.0001.

Since M-MDSCs were expanded in the peripheral tissues but not within the bone marrow, we argued that transcriptomic changes within the expanded antecedents of M-MDSCs in bone marrow would identify TTP-dependent mRNAs engaged in M-MDSC mobilization. We analyzed the scRNA-seq data sets focusing on the myeloid progenitor populations. After selecting the myeloid progenitor population, including the early monocyte population (**Figure 6A**), re-clustering was performed, and each cell type was identified and annotated according to the markers expressed in each sub-cluster (**Figures 6B, C**). Similar to the flow cytometry results, the transcript profile of common monocyte progenitor (cMoP)/MP, early monocyte, and early MDSC were increased in cTTPKO and TTPKO mice compared to the control mice (**Figure 6D**).

To determine whether the expanded M-MDSCs and the ability of their progenitors to mobilize from the bone marrow into the blood and differentiate into M-MDSCs in the periphery, we analyzed the expression levels of key receptors mediating mobilization, notably *Ccr2* (30, 31). In cTTPKO or TTPKO mice, *Ccr2* expression was high in M-MDSC progenitor cells including the cMoP/MP cluster, but no significant change in *Cxcr2* expression was observed in the GP cluster except the early MDSC cluster (**Figure 6E**). Moreover, there was no significant difference in the expression of *Ccr2* in the M-MDSC population (**Figure 6F**), suggesting the possibility that cMoP/MP, and early MDSC, but not M-MDSCs, could exit the bone marrow. Since chemokines are known to recruit immune cells by activating receptors on their surface, we measured prominent chemokines in the serum (32, 33). As shown in **Figure 6G**, cTTPKO and TTPKO mice had significantly higher levels of CXCL2, a major ligand of CXCR2, and CCL2, a major ligand of CCR2, compared to the controls. There was little expression of egress-associated mRNAs from the stromal component within the bone marrow and no significant changes in myeloid cell populations cell cycling was observed in the scRNAseq datasets (**Figures S5, S6**). These results suggest that the CCR2-CCL2 axis contributes to increased MDSCs (potentially both PMN- and M-MDSCs), due to the lack of TTP as would be observed in age-related TTP decline. To determine whether chemokines were increased in the serum of aged mice as in cTTPKO or TTPKO mice, sera of aged mice were analyzed. Similar to serum levels found in cTTPKO or TTPKO mice, the concentrations of target chemokines were significantly increased in aged mice (**Figure 6H**). In addition to CCL2, CXCL2, IL-6, and TNFα were elevated in cTTPKO, TTPKO (**Figure 6G**) and aged mice (cTTPKO/TTPKO), respectively. Collectively, these data help to advance our understanding of the age-related phenotype that accompanies TTP decline.

## Discussion

TTP, an RNA-binding protein that destabilizes target mRNA and promotes degradation, regulates the fate of myeloid cell populations, including MDSCs. In aged myeloid cells, the TTP expression levels decrease (16), and the balance of myeloid cell differentiation is shifted towards innate immunity, since the adaptive immune response declines due to impairment of lymphocyte subsets and thymic involution (34). Aging not only modulates the balance of immune cells, but also affects the development and maturation within the bone marrow. With inflammaging, cytokines such as TNF-α and IL-6 are excessively produced, leading to age-related myelopoiesis (35–37). Because of the inflammatory phenotype seen in TTP-deficient mice, initial studies primarily reported on Gr-1^+^ granulocytes, where the number of mature granulocytes increased in the bone marrow of adult mice deficient in TTP (14), and that CD11b^+^Gr-1^+^ cells (a bulk MDSC phenotype) accumulated in the bone marrow and spleen of young TTPKO mice (38). Since TTP regulates the inflammatory cytokine/chemokine signatures associated with inflammaging, we explored mechanisms of MDSC expansion that accompany TTP-related decline with aging.

With age-related TTP decline, as modeled in TTP global and myeloid-specific null mice, we showed a concomitant expansion of M-MDSCs and transcriptomic changes in the CCR2-CCL2 axis within bone marrow myeloid progenitor populations that may contribute to their mobilization from bone marrow and peripheral expansion. These changes do not exclude the possibility that other cellular or intracellular mechanistic changes in the aged bone marrow microenvironment contribute towards increased myelopoiesis. However, chemokine expression in stromal lineage cell populations within the bone marrow (Supplemental Data) indicates low expression of *Ccl2 and Cxcl2*. These data help to support the conclusion that the non-myeloid compartment does not significantly contribute to the chemokines driving M-MDSC expansion in the TTP engineered mice. In addition, we did not age these TTP-engineered mice to determine whether these changes are or are not influenced by age. Nevertheless, we found that the loss of TTP in the myeloid progenitor compartment was sufficient to phenocopy the global loss of TTP, suggesting that cell-intrinsic mechanisms contribute towards age-related M-MDSC expansion.

We observed that there was no difference in the population of M-MDSCs in the bone marrow when TTP was deficient, whereas the percentage was consistently higher in the spleen and MLN. Although there was no change in the M-MDSCs in the bone marrow, detailed flow cytometric analyses of the myeloid cell progenitor populations revealed an increase in both cTTPKO and TTPKO mice. In both cTTPKO and TTPKO mice, the population with the most notable change in myeloid cell progenitor populations was the total GMP. These findings are consistent with published data indicating that the GMP population was increased in TTPKO mice (38). However, it appears that an increase in the GMP population does not support only an increase in granulocytes, as others have observed. In the present study, the oligopotent GMP and MP populations of cTTPKO and TTPKO mice increased, whereas the GP population increased only in TTPKO mice. Also, in both cTTPKO and TTPKO mice, the oligopotent GMP population increased the most among the total GMP population, but the significant increase in the MP to GP ratio also indicates that total GMP is more biased towards MP than GP fate, and the oligopotent GMP population is also likely to be the same. This result is consistent with a previous report that HSPCs dramatically egress into the circulation under stress condition or situations requiring increased hematopoiesis (39).

Although there were many similar trends between the flow cytometry and scRNA-seq results in this study, some discrepancies were observed. This discrepancy mainly occurred in the cluster that occupies a small proportion of the total cell population, which appears to result from overlapping CD markers used to distinguish HSPC lineage cells. As other studies have also reported discrepancies for similar populations (25, 40), opportunities for further improvement, particularly in distinguishing small populations, should be highlighted with the importance of growing single-cell use with concurrent flow cytometry data sets.

The CC-chemokine ligand 2 (CCL2; also known as MCP-1) attracts cells through activation of CCR2, a cognate receptor expressed on the surface of monocyte populations (41). CCL2 expression is induced by pro-inflammatory cytokines and maintained high in serum and inflamed tissues. This is a key trigger for CCR2-expressing monocyte populations to be mobilized into the circulation or inflamed tissue. Moreover, CCL2 is known to differentiate and mature monocytes from M-MDSCs, as well as influence their mobilization (42, 43). We show that expression of *Ccr2* was highly expressed in the cMoP/MP population of TTPKO or cTTPKO mice. These observations strongly suggest that cMoP/MP, a progenitor cell of M-MDSCs in TTPKO or cTTPKO mice, is differentiated into M-MDSCs in the periphery after mobilization into the circulation and increased in the spleen and mLN. Moreover, CCL2 has been reported as one of the targets of TTP, and when TTP is absent, CCL2 remains high throughout the body including blood, supporting the differentiation of MPs into M-MDSCs after mobilization (44–46). These transcriptomic data are supported by our data that CCL2 was significantly higher in the serum of cTTPKO and TTPKO mice, as well as aged WT mice. Consistent with these observations are other studies showing increased CCR2 expression on monocytic cells in the bone marrow and that their subsequent mobilization is TNF-dependent (47). Additional studies are needed to determine whether the myeloid cells were directly increased by TTP deficiency or indirectly by increase cytokines within the bone marrow microenvironment.

In summary, we found that as TTP expression within myeloid cell populations decreases with aging, there is an increased expansion of total GMP and MP cells, likely direct precursors of M-MDSCs, in both cTTPKO and TTPKO mice. A detailed transcriptomic analysis of cTTPKO and TTPKO bone marrow revealed the expression of CCR2, a crucial monocyte progenitor mobilization signal, was dramatically increased along with elevated levels of serum CCL2, a major ligand of CCR2, suggesting that M-MDSC progenitor cells mobilized due to alterations in the CCR2-CCL2 axis with reduced TTP expression as observed in aging. Collectively, these studies demonstrate that TTP may play a pivotal role in regulating aging-associated myelopoiesis by controlling the expansion of specific subpopulations of myeloid progenitor cells through disruption of the chemokine CCR2-CCL2 axis.

## Data availability statement

The data presented in the study are deposited in the NCBI Gene Expression Omnibus (GEO) repository, accession number GSE210910.

## Ethics statement

The animal studies were reviewed and approved by the Institutional Animal Care and Use Committee at the University at Buffalo.

## Author contributions

KHK performed experiments and wrote the manuscript. EK, LZ, RT, performed experiments and edited the manuscript. SA, KLK conceptualized and designed all experiments and edited the manuscript. PB and YA provided mouse strains and edited the manuscript. BT and KS provided data interpretation and edited the manuscript. JB and NL performed bioinformatic analyses of RNAseq data and edited the manuscript. All authors contributed to the article and approved the submitted version.

## Funding

This work was supported by the National Institutes of Health (NIH) grants, R01DE028258 (to KLK), R01DE028258S1 (KLK, SA), K18DE029526 (KLK) and by the Basic Science Research Program through the National Research Foundation of Korea (NRF) funded by the Ministry of Education, NRF-2021R1A6A03044546 (to KHK). It was also supported by the Indian Trail Foundation (BRT) and in part by the Intramural Research Program of the NIEHS, NIH (YA and PB). Flow cytometry data in this manuscript was supported by the Optical Imaging and Analysis Facility at the University at Buffalo.

## Conflict of interest

The authors declare that the research was conducted in the absence of any commercial or financial relationships that could be construed as a potential conflict of interest.

## Publisher’s note

All claims expressed in this article are solely those of the authors and do not necessarily represent those of their affiliated organizations, or those of the publisher, the editors and the reviewers. Any product that may be evaluated in this article, or claim that may be made by its manufacturer, is not guaranteed or endorsed by the publisher.
