# Supplementary material for: Tristetraprolin limits age-related expansion of myeloid-derived suppressor cells
Source: Front Immunol. 2022 Oct 3;13:1002163. doi: 10.3389/fimmu.2022.1002163 (PMC9573970; doi:10.3389/fimmu.2022.1002163)
Supplement: Supplementary file 1 [file Presentation_1.pptx]

## Slide 1
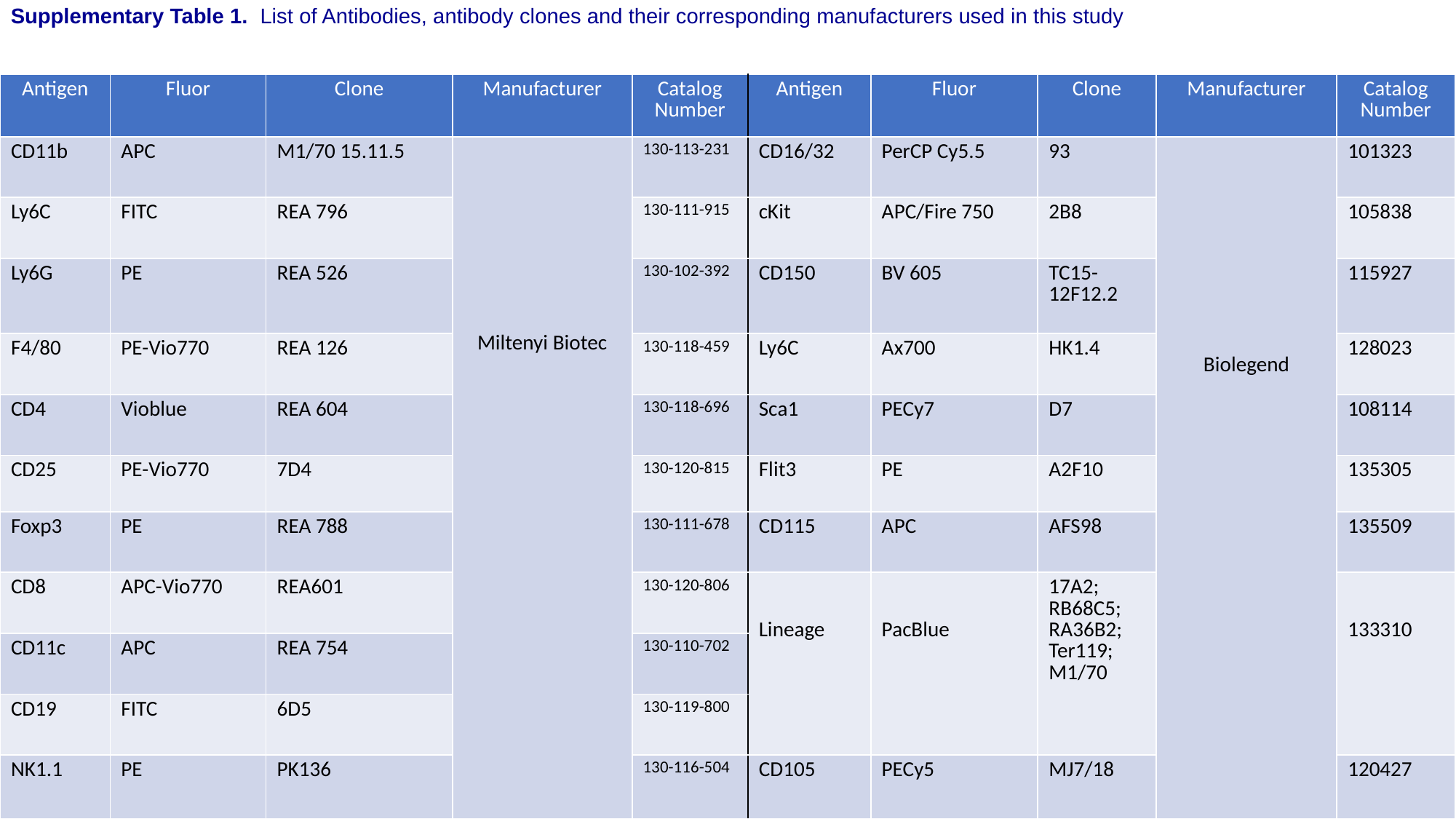

Supplementary Table 1. List of Antibodies, antibody clones and their corresponding manufacturers used in this study
| Antigen | Fluor | Clone | Manufacturer | Catalog Number | Antigen | Fluor | Clone | Manufacturer | Catalog Number |
| --- | --- | --- | --- | --- | --- | --- | --- | --- | --- |
| CD11b | APC | M1/70 15.11.5 | Miltenyi Biotec | 130-113-231 | CD16/32 | PerCP Cy5.5 | 93 | Biolegend | 101323 |
| Ly6C | FITC | REA 796 | | 130-111-915 | cKit | APC/Fire 750 | 2B8 | | 105838 |
| Ly6G | PE | REA 526 | | 130-102-392 | CD150 | BV 605 | TC15-12F12.2 | | 115927 |
| F4/80 | PE-Vio770 | REA 126 | | 130-118-459 | Ly6C | Ax700 | HK1.4 | | 128023 |
| CD4 | Vioblue | REA 604 | | 130-118-696 | Sca1 | PECy7 | D7 | | 108114 |
| CD25 | PE-Vio770 | 7D4 | | 130-120-815 | Flit3 | PE | A2F10 | | 135305 |
| Foxp3 | PE | REA 788 | | 130-111-678 | CD115 | APC | AFS98 | | 135509 |
| CD8 | APC-Vio770 | REA601 | | 130-120-806 | Lineage | PacBlue | 17A2; RB68C5; RA36B2; Ter119; M1/70 | | 133310 |
| CD11c | APC | REA 754 | | 130-110-702 | | | | | |
| CD19 | FITC | 6D5 | | 130-119-800 | | | | | |
| NK1.1 | PE | PK136 | | 130-116-504 | CD105 | PECy5 | MJ7/18 | | 120427 |

## Slide 2
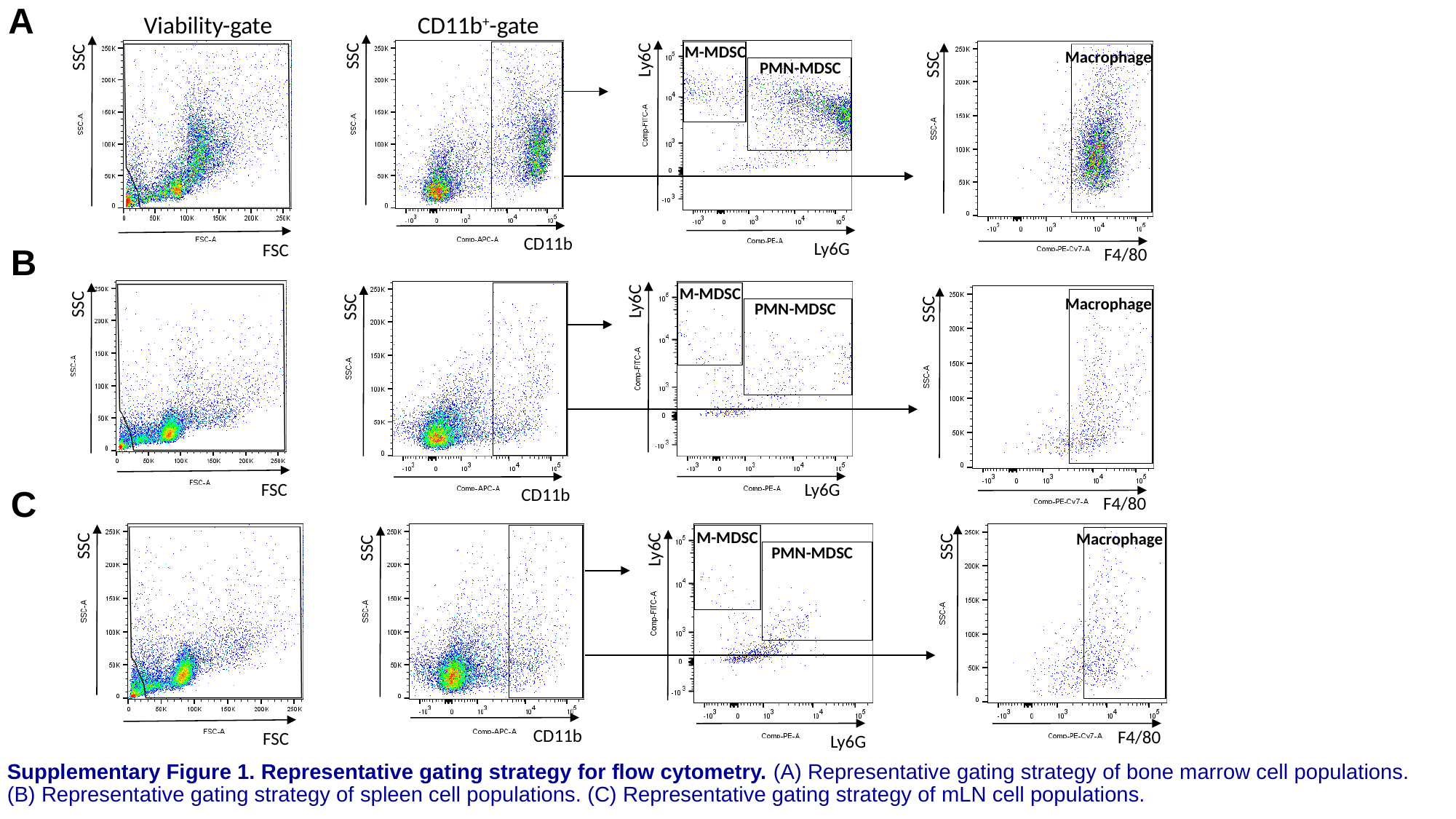

A
CD11b+-gate
Viability-gate
M-MDSC
Ly6C
PMN-MDSC
Ly6G
SSC
SSC
SSC
CD11b
FSC
F4/80
Macrophage
B
M-MDSC
Ly6C
SSC
SSC
SSC
PMN-MDSC
FSC
Ly6G
CD11b
F4/80
Macrophage
C
M-MDSC
SSC
SSC
SSC
Ly6C
PMN-MDSC
CD11b
F4/80
FSC
Ly6G
Macrophage
Supplementary Figure 1. Representative gating strategy for flow cytometry. (A) Representative gating strategy of bone marrow cell populations. (B) Representative gating strategy of spleen cell populations. (C) Representative gating strategy of mLN cell populations.

## Slide 3
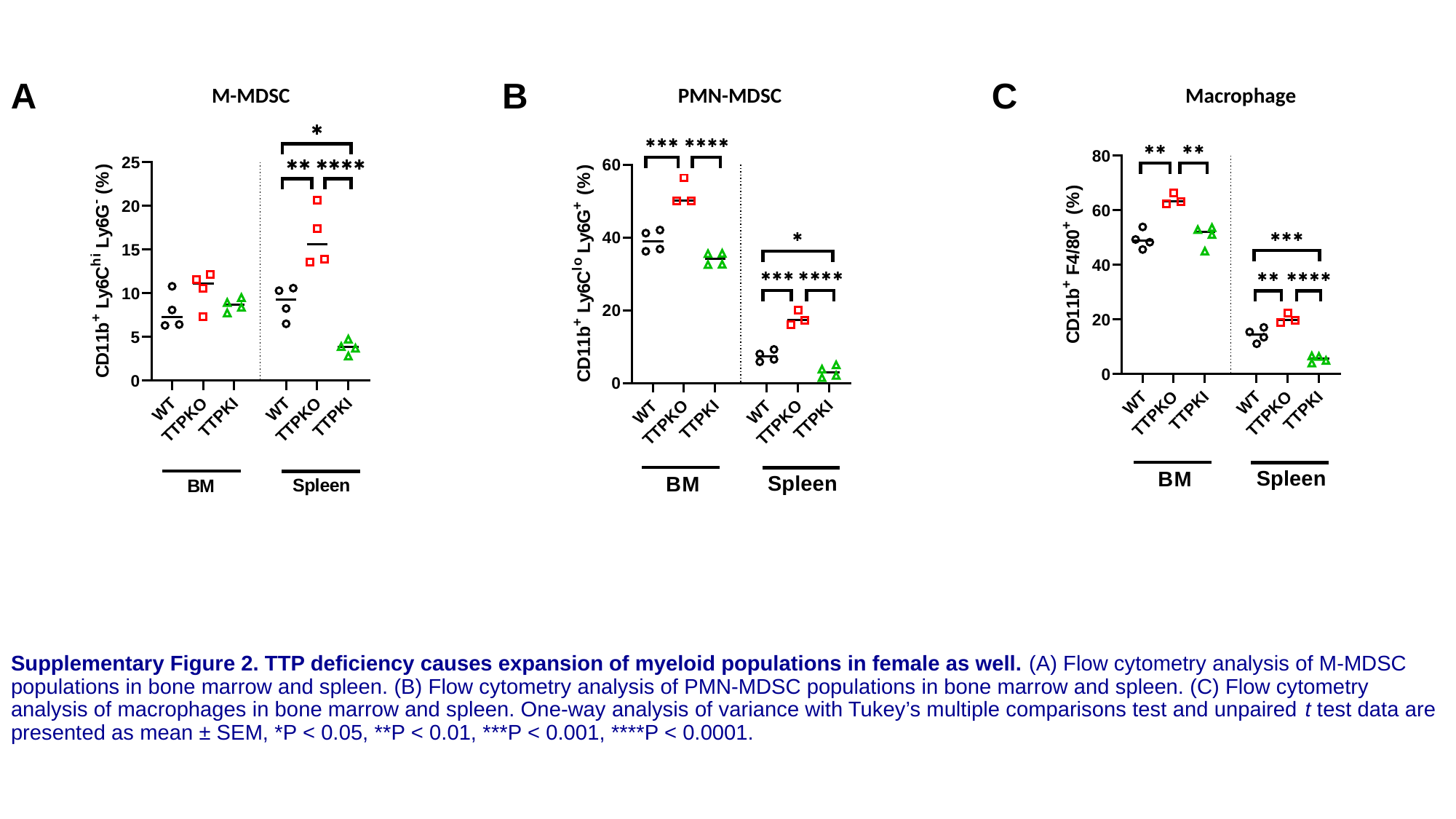

A
B
C
M-MDSC
PMN-MDSC
Macrophage
Supplementary Figure 2. TTP deficiency causes expansion of myeloid populations in female as well. (A) Flow cytometry analysis of M-MDSC populations in bone marrow and spleen. (B) Flow cytometry analysis of PMN-MDSC populations in bone marrow and spleen. (C) Flow cytometry analysis of macrophages in bone marrow and spleen. One-way analysis of variance with Tukey’s multiple comparisons test and unpaired t test data are presented as mean ± SEM, *P < 0.05, **P < 0.01, ***P < 0.001, ****P < 0.0001.

## Slide 4
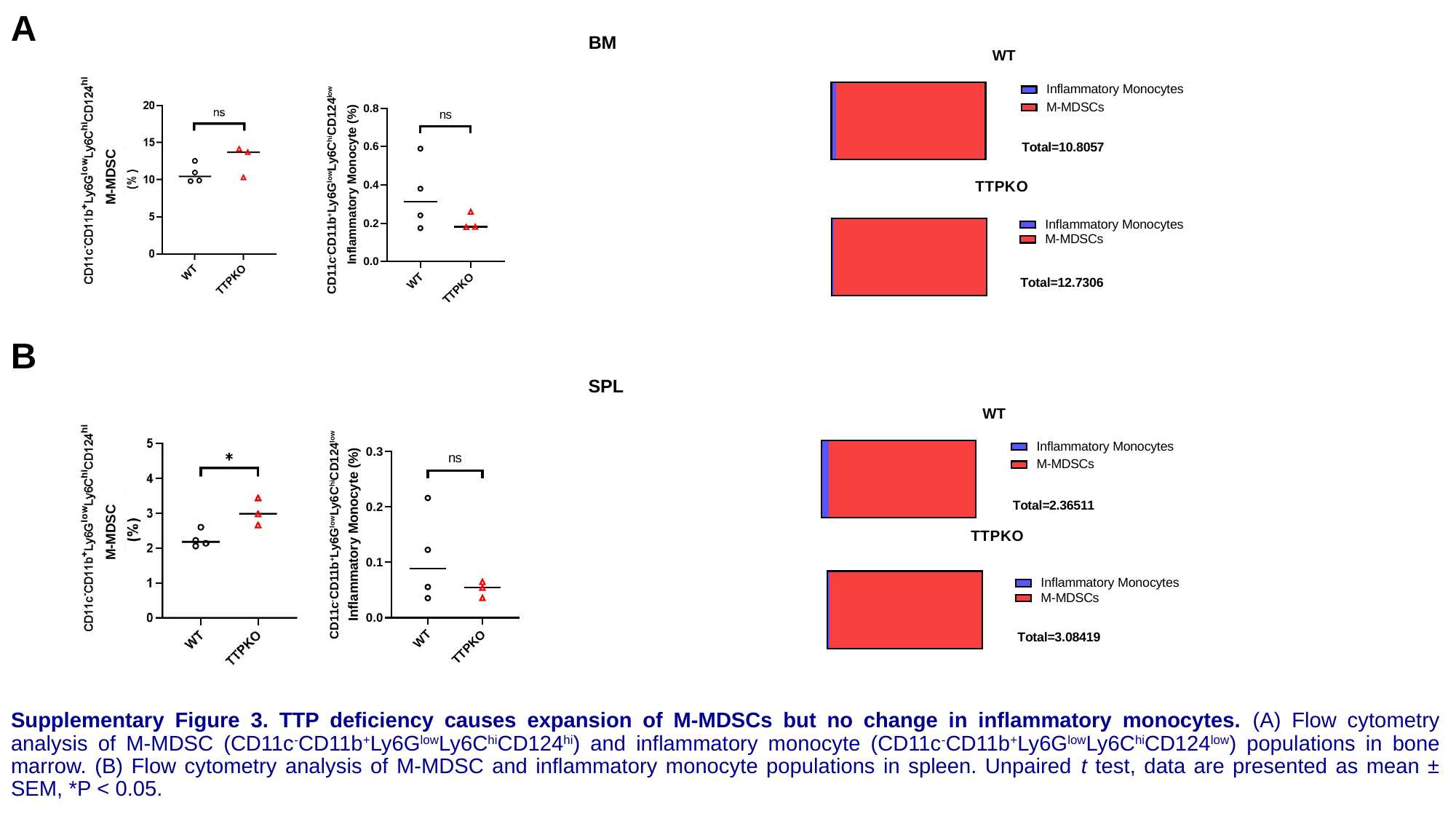

A
BM
M-MDSC
CD11c-CD11b+Ly6GlowLy6ChiCD124low
B
SPL
M-MDSC
CD11c-CD11b+Ly6GlowLy6ChiCD124low
Supplementary Figure 3. TTP deficiency causes expansion of M-MDSCs but no change in inflammatory monocytes. (A) Flow cytometry analysis of M-MDSC (CD11c-CD11b+Ly6GlowLy6ChiCD124hi) and inflammatory monocyte (CD11c-CD11b+Ly6GlowLy6ChiCD124low) populations in bone marrow. (B) Flow cytometry analysis of M-MDSC and inflammatory monocyte populations in spleen. Unpaired t test, data are presented as mean ± SEM, *P < 0.05.

## Slide 5
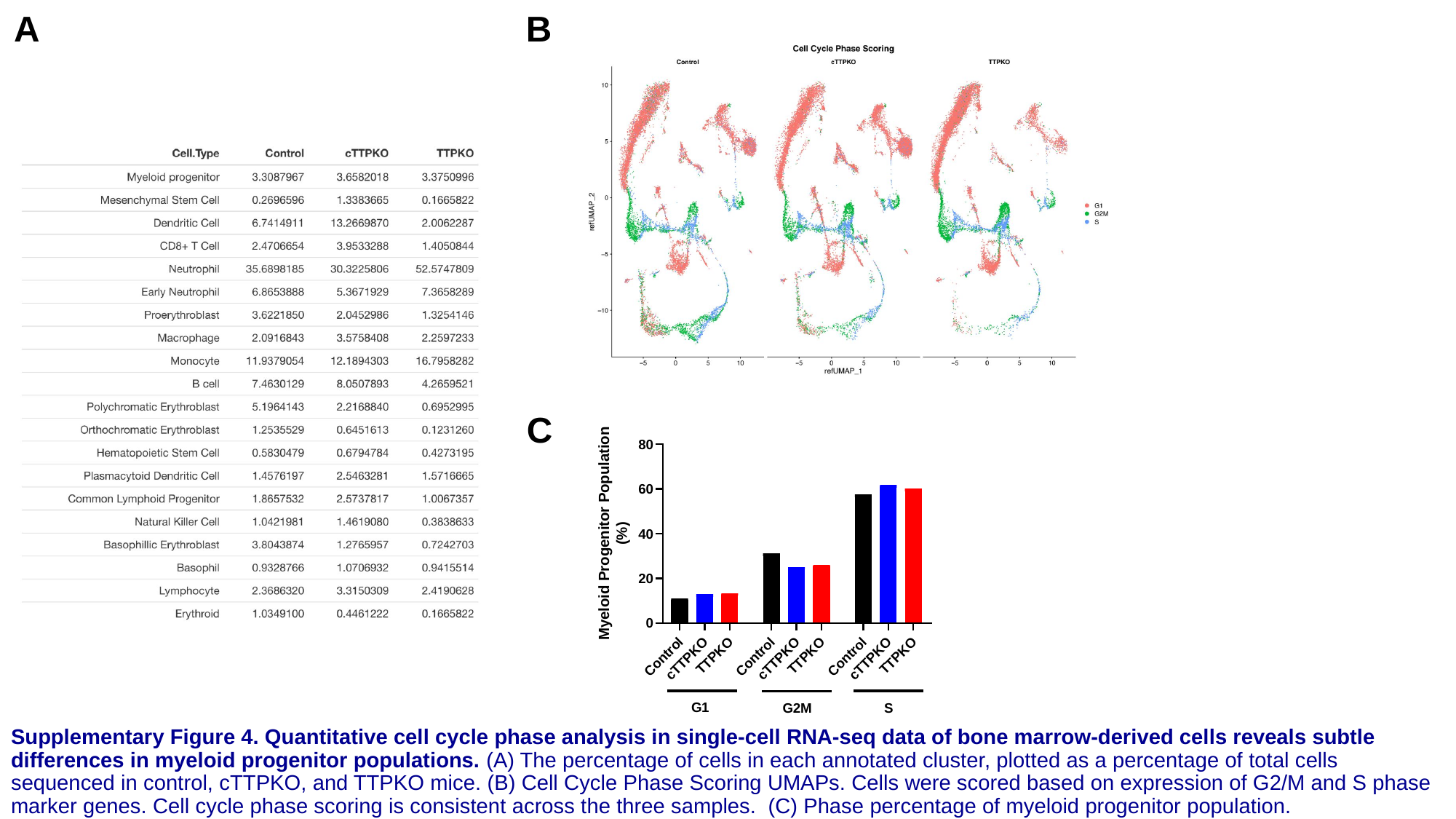

A
B
C
Supplementary Figure 4. Quantitative cell cycle phase analysis in single-cell RNA-seq data of bone marrow-derived cells reveals subtle differences in myeloid progenitor populations. (A) The percentage of cells in each annotated cluster, plotted as a percentage of total cells sequenced in control, cTTPKO, and TTPKO mice. (B) Cell Cycle Phase Scoring UMAPs. Cells were scored based on expression of G2/M and S phase marker genes. Cell cycle phase scoring is consistent across the three samples. (C) Phase percentage of myeloid progenitor population.

## Slide 6
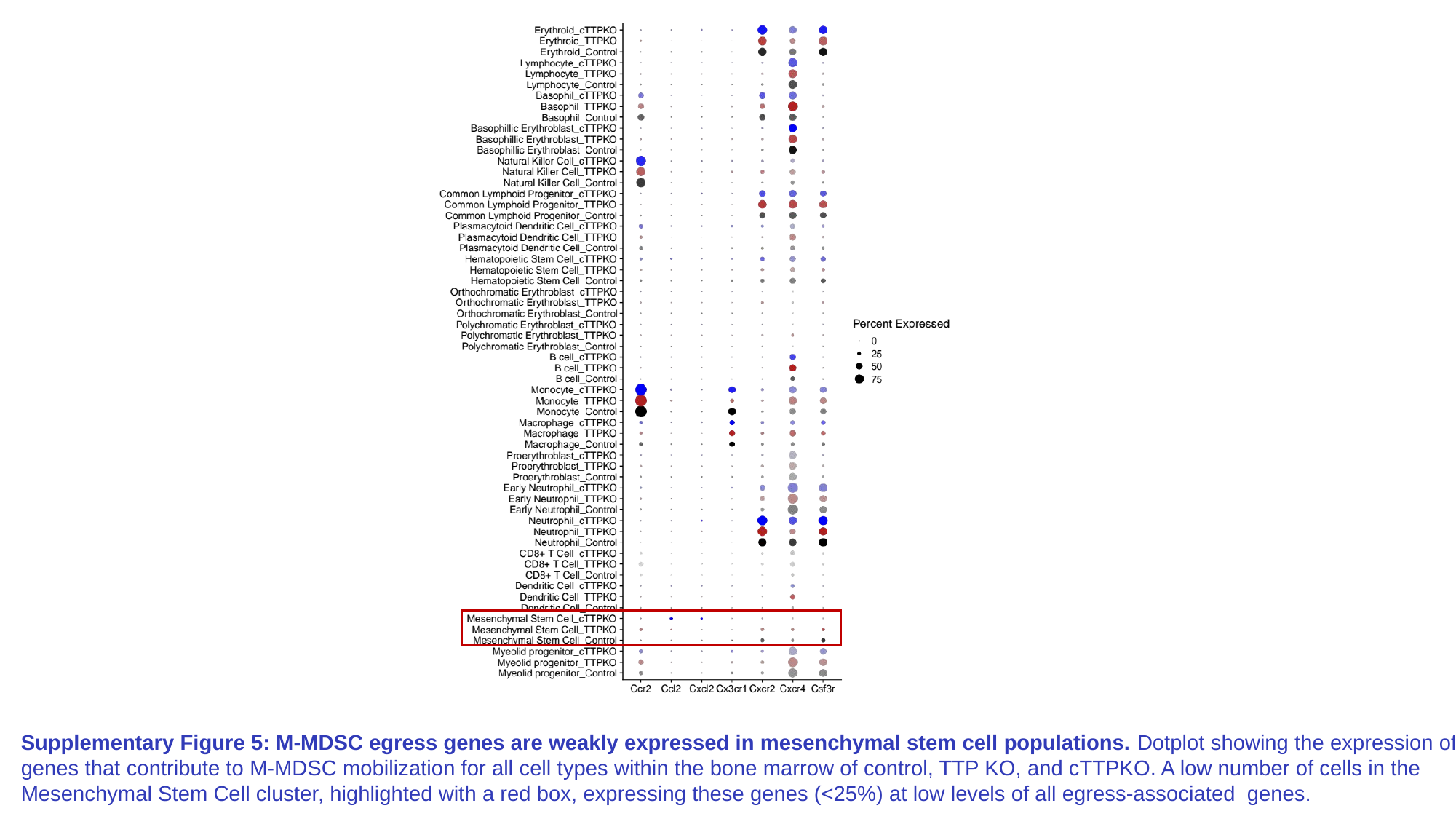

Supplementary Figure 5: M-MDSC egress genes are weakly expressed in mesenchymal stem cell populations. Dotplot showing the expression of genes that contribute to M-MDSC mobilization for all cell types within the bone marrow of control, TTP KO, and cTTPKO. A low number of cells in the Mesenchymal Stem Cell cluster, highlighted with a red box, expressing these genes (<25%) at low levels of all egress-associated genes.
